# Negative pressure pulmonary edema and hemorrhage after near fatal suffocation in an infant: a case report

**DOI:** 10.1186/s13052-025-02015-6

**Published:** 2025-06-07

**Authors:** Valentina Tonazzo, Chiara Po’, Silvia Bertolo, Valentina Agnese Ferraro, Stefania Zanconato, Stefano Martelossi, Silvia Carraro

**Affiliations:** 1 Unit of Pediatric Allergy and Respiratory Medicine Women’s and Children’s Health Department, University of Padova, Via Giustiniani 3, Padova, 35128 Italy; 2 Pediatric Unit, AULSS 2 Marca Trevigiana, Ca’ Foncello Hospital, Treviso, Italy; 3 Radiology Unit, AULSS 2 Marca Trevigiana, Ca’ Foncello Hospital, Treviso, Italy

**Keywords:** Case report, Pulmonary hemorrhage, Negative pressure pulmonary edema, Pediatric pulmonology

## Abstract

**Background:**

Pulmonary hemorrhage is rare but potentially life-threatening in children. Many causes are usually described, as cardiogenic, infective or immune. Pulmonary hemorrhage related to negative pressure pulmonary edema (NPPE) is uncommon in the pediatric population and there is limited literature about it. This is one of the few case reports regarding NPPE in infants presenting with pulmonary hemorrhage.

**Case presentation:**

We describe the story of a 6-weeks-old boy who presented epistaxis and hemoptysis associated with symptoms related to NPPE after near fatal suffocation. Radiological findings were consistent with alveolar hemorrhage. Supportive therapy was performed, with clinical recovery within a few days and radiological normalization within one month.

**Conclusion:**

NPPE associated with pulmonary hemorrhage is a dramatic condition but usually has a quick recovery with just supportive therapy. The aim of our report is to increase the awareness and emphasizes the importance of including this entity in the differential diagnosis of pulmonary hemorrhage in children with a suspicious anamnestic history of upper airway obstruction.

## Background

Pulmonary hemorrhage is rare but potentially life-threatening in pediatric population [1]. Etiopathogenesis is usually related to congenital heart disease, prematurity/premature lung disease, lung disorders such as cystic fibrosis, respiratory infections, coagulopathies or, unfrequently, nonaccidental trauma [2–4]. In older children and adults also immune-related diseases such as vasculitis or collagenopathies and cancers are reported as possible causes, whereas idiopathic pulmonary hemorrhage is described at any age, as a diagnosis of exclusion [5, 6].

Very little is known about epidemiology, incidence and outcomes of pulmonary hemorrhage associated with negative pressure pulmonary edema (NPPE). NPPE is a well-known entity in adults especially as a postoperative and anaesthetic complication [7, 8], while it is less described in children, due to the lack of awareness and limited number of cases reported in literature.

## Case presentation

A 6-weeks-old previously healthy boy was admitted to the Paediatric Emergency Department after an episode of epistaxis and profuse hemoptysis. He was sleeping on his mother’s chest after being breastfed, with his 2 years old brother lying upon him, when his father heard a moaning noise. He noticed that the baby had a noisy breathing and that he had epistaxis. Right after, the infant profusely vomited bloody secretions mixed with milk.

The infant was born at term and had a normal growth. His past medical history was silent except for mild rhinitis and cough in the previous two days. Family history was silent as well.

On admission to the Emergency Department, the child was pale and slightly hypotonic and presented tachycardia, tachypnea and dyspnea with a room air oxygen saturation of 92% and coarse crackles on chest auscultation. The remaining physical examination was normal.

Laboratory studies revealed normocytic anaemia (haemoglobin level 7.5 g/dl) and increased serum creatine phosphokinase and myoglobin. The coagulation profile was normal. Microbiological analysis of blood and nasal swab was negative, as was the search for toxic substances in urine.

Echocardiography and cerebral CT and MRI were unremarkable, as well as whole-body bone scintigraphy and retinal examination performed to exclude maltreatment as a possible cause of the bleeding.

**Fig. 1:**
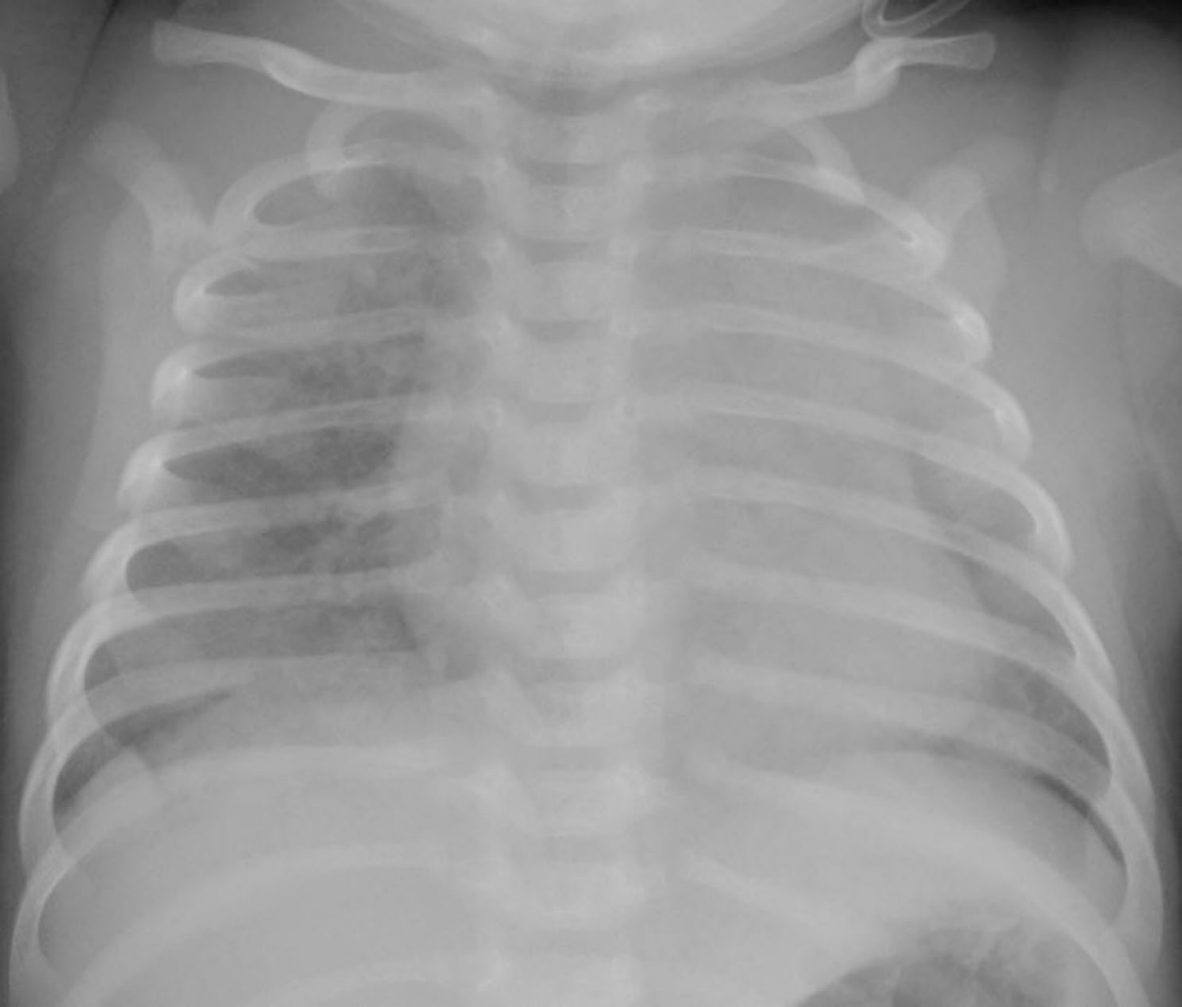
Chest X-ray

**Fig. 2:**
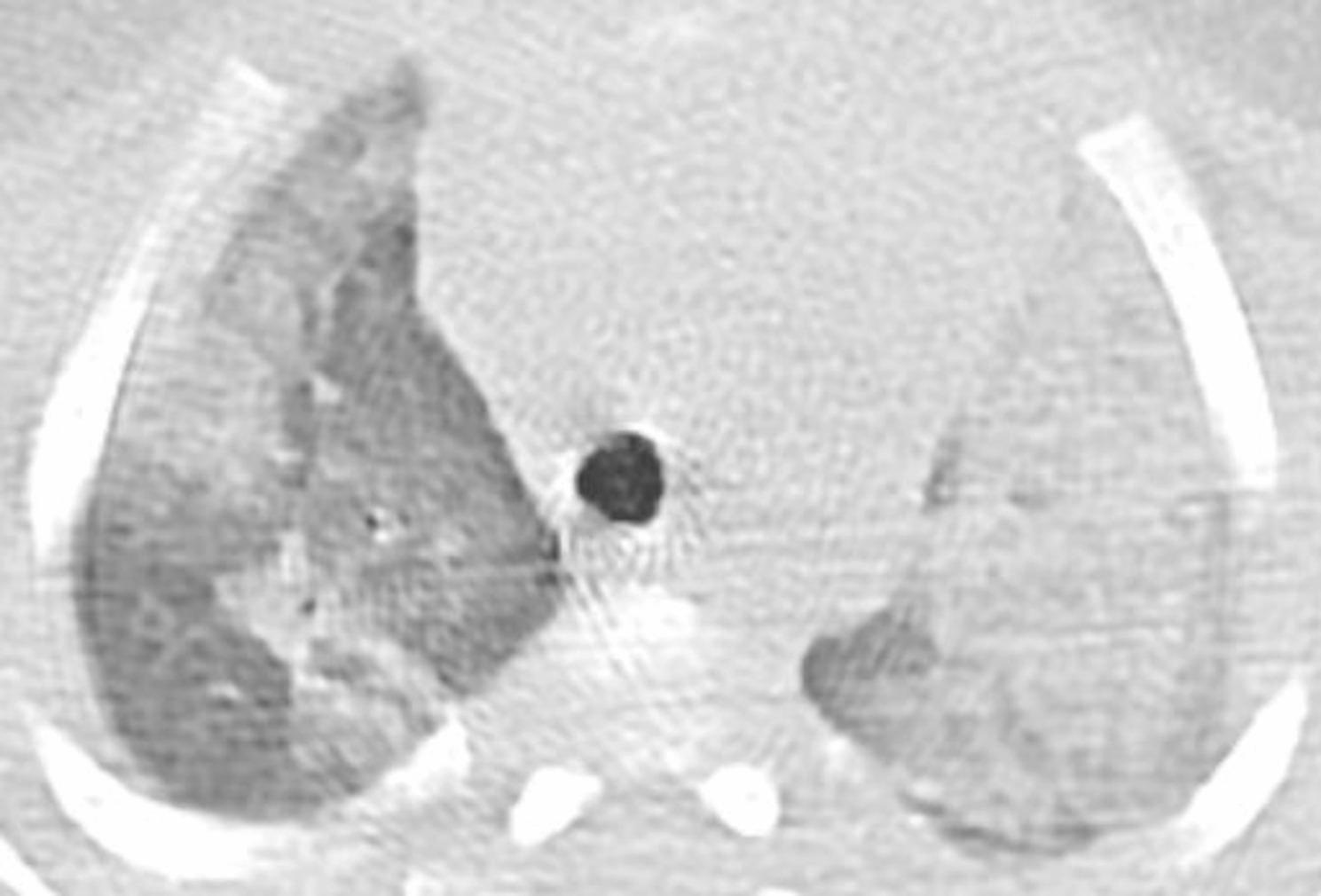
CT at the level of trachea

**Fig. 3:**
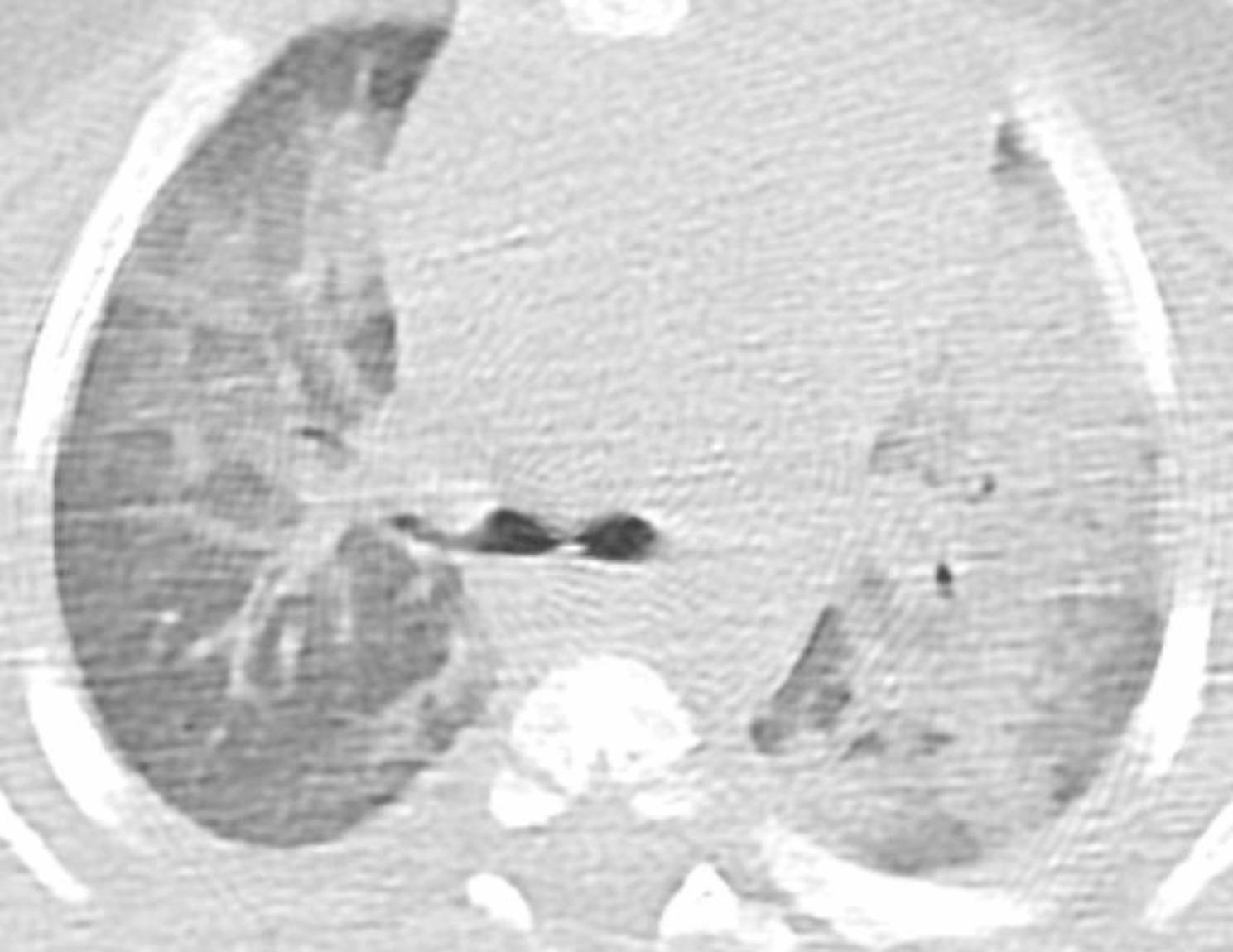
CT at the level of carina

**Fig. 4:**
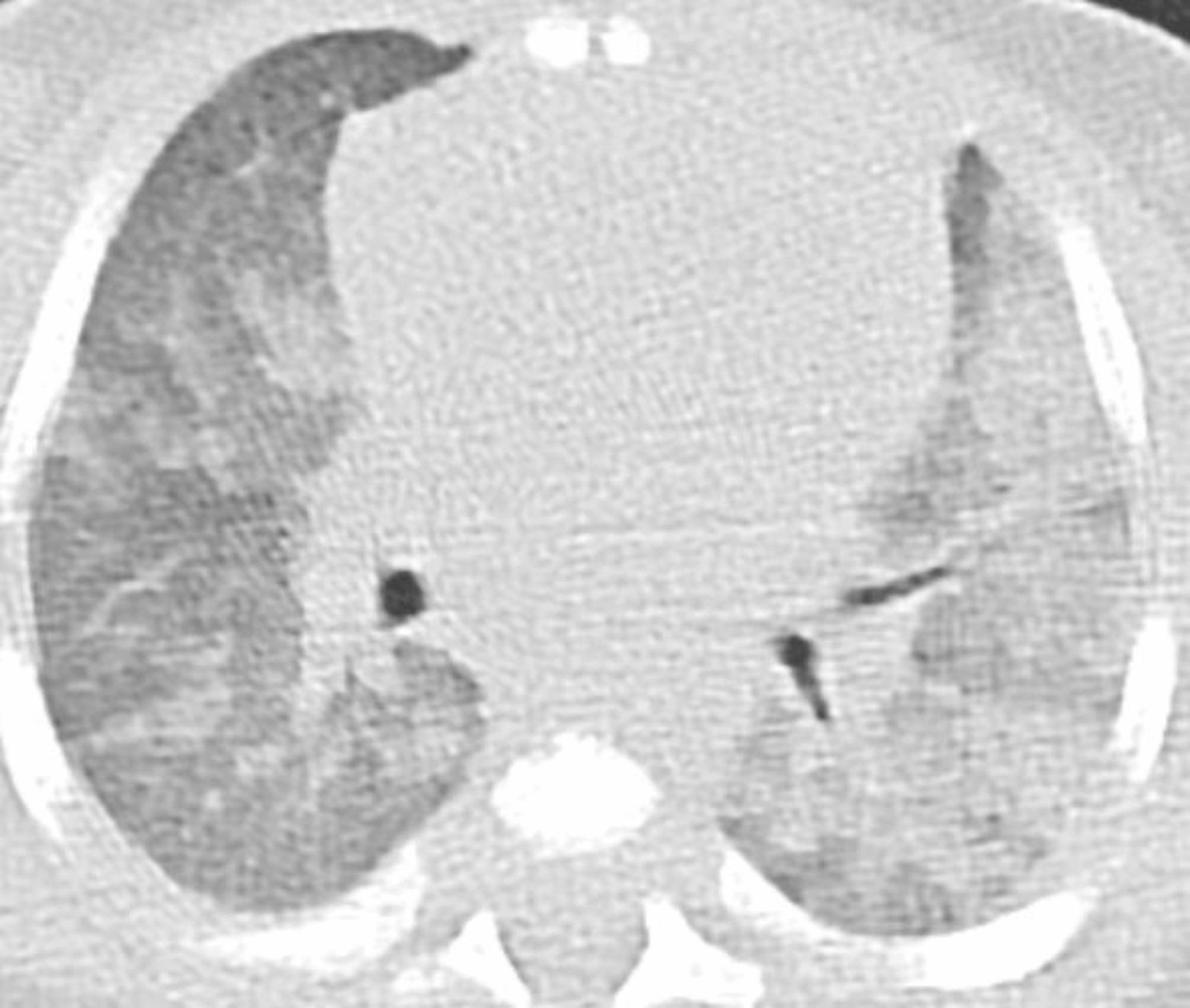
CT at the level of bronchial branches

The chest X-ray revealed diffuse interstitial opacities (Fig. 1). The chest CT showed diffuse patchy bilateral alveolar opacities and consolidation areas as well as ground-glass opacities, which were more evident in the left pulmonary lobe (Figs. 2, 3 and 4).

The infant received blood transfusion, non-invasive ventilatory support for 24 h and antibiotic treatment with an intravenous third-generation cephalosporin, with complete recovery within a few days without any further bleeding or symptoms. Antibiotic therapy was changed to amoxicillin-clavulanic acid after three days and continued for a total of seven days. One month later a chest CT scan was performed, which revealed normal pulmonary architecture.

## Discussion and conclusions

Negative pressure pulmonary edema (NPPE), also called post-obstructive pulmonary edema, was first described in animal models in 1927 [9], while the first report of pulmonary edema associated with upper airway obstruction was published in 1973 [10]. Since then, several case reports and series were reported in literature but true incidence is still unknown.

NPPE is triggered when a forced inspiratory effort is made versus an obstructed upper airway. This leads to a markedly negative intrathoracic pressure, which increases venous return to the right ventricle. Because of ventricular interdependence, there is a shift of intraventricular septum to the left side which decreases left ventricular compliance while increasing left ventricular end-diastolic pressure and, subsequently, pulmonary capillary transmural pressure, with fluid leakage in interstitial spaces. When the abnormal amount of fluid overwhelms the re-absorptive capacity of the pulmonary lymphatic channels, the result is extravasation of fluid into the alveoli causing pulmonary edema [11, 12].

Another factor implicated in the pathogenesis of NPPE is the absence of airflow and the resulting lack of alveolar gas exchange due to upper airways obstruction. Consequent hypoxia and hypercarbia are responsible for pulmonary vasoconstriction and catecholamine discharge, which in turn leads to systemic vasoconstriction and increased left ventricle afterload [11, 13]. This results in further elevation of pulmonary capillary transmural stress till disruption of the alveolar-capillary membrane, with impairment of barrier function and extravasation of red blood cells into alveolar spaces [14]. Furthermore, it has been proposed that blood coming from alveoli into the upper airways may favour laryngospasm adding onto airway obstruction in a vicious cycle [15].

NPPE has rapid onset of symptoms, during upper airways obstruction or shortly after the relief of the obstruction [16]. Clinical presentation may vary from dyspnea, progressive cyanosis, anxiety and increased work of breathing to severe cases of respiratory failure [8, 17]. Sometimes peripheral hypoperfusion, bradycardia and hypotension are described as the result of myocardial depression due to hypoxia and acidosis [15]. Crackles are usually appreciated on pulmonary auscultation while the radiographic image is of alveolar filling [12, 18].

Some of those clinical aspects were presented by the patient described in our case report. He presented with dyspnea, cyanosis and crackles on chest auscultation and needed a non-invasive respiratory support. The radiological findings of diffuse bilateral alveolar opacities and ground-glass opacities and consolidation reported in our case are consistent with pulmonary edema similarly to other cases of NPPE published recently [18].

Given anamnestic data, NPPE in our patient could be explained by the obstruction of upper airways due to prone sleeping position with thorax caught under the brother’s body.

As reported in a recent review of the literature, in the past two decades there has been a shift in the etiology of NPPE in pediatric population [18]. Historically, NPPE was caused by glottic or subglottic obstruction due to acute upper airway infection like croup or epiglottitis [19], which became less common after the introduction of the Hib vaccine in 1993. Recently, there have been anecdotal cases of NPPE in children, mainly related to postextubation laryngospasm or airway obstruction by secretion or foreign body inhalation [18, 20]. Few reports also describe asphyxiation by near strangulation and choking as the cause of NPPE [15, 21, 22], as shown in our patient.

What made the diagnosis difficult in this case was the finding of epistaxis and hemoptysis and radiological images suitable for alveolar hemorrhage as well as pulmonary edema. Most of the papers in literature report just pink frothy secretion from the mouth as associated with NPPE [8, 16].

Pulmonary haemorrhage due to blunt trauma was considered as a differential diagnosis in our patient, but it seemed unlikely given the small extent of the rib fractures and the absence of other signs of trauma. Coagulation disorders, respiratory or systemic infections, heart disease or child abuse were also considered as possible causes of diffuse alveolar hemorrhage, but they were ruled out by the unremarkable biochemical, instrumental, and microbiological analyses.

Reviewing literature, the first mention of pulmonary hemorrhage associated with NPPE dates back to 1999 [21], tracing the physiopathological mechanism of alveolar hemorrhage to pulmonary capillary transmural stress mentioned above. After that, some case reports described infants presenting with pulmonary hemorrhage and NPPE as a sequelae of accidental asphyxiation, [18, 23]. It has also been known for years that deliberate or accidental suffocation may lead to both nasal and intrapulmonary hemorrhage [24]. A review by Hey published in 2008 found eighty-eight papers describing bleeding from mouth, nose and larynx after an episode of sudden upper airways obstruction [23] and a systematic review published in 2016 by Rees et al. confirmed the possible association between epistaxis and asphyxia in children aged < 2 years [25], such as our patient.

Although it may initially be life-threatening, NPPE is usually a benign condition that resolves in 24–48 h with only supportive treatment consisting of non-invasive or invasive positive pressure ventilation, oxygen supplementation and diuretics if necessary [16].

As shown in our story, diagnosis is possible considering clinical presentation, radiological findings and time course, but primary suspicion should come from the anamnestic scenario. In the case of pulmonary hemorrhage due to acute upper airway obstruction, being aware of and recognizing NPPE allows the prompt initiation of a proper treatment and prevents unnecessary investigations and iatrogenic complications related to the treatment indicated for other forms of pulmonary hemorrhage.

## Data Availability

The data used to write this report are available from the corresponding author upon reasonable request.

## Abbreviations

CT: Computed Tomography
MRI: Magnetic Resonance Imaging
NPPE: Negative Pressure Pulmonary Hemorrhage
